# State of the Art in Pediatric Anesthesia: A Narrative Review about the Use of Preoperative Time

**DOI:** 10.3390/jpm14020182

**Published:** 2024-02-06

**Authors:** Fabio Sbaraglia, Christian Cuomo, Filomena Della Sala, Rossano Festa, Rossella Garra, Federica Maiellare, Daniela Maria Micci, Domenico Posa, Cecilia Maria Pizzo, Angela Pusateri, Michelangelo Mario Spano, Monica Lucente, Marco Rossi

**Affiliations:** Department of Anesthesia and Intensive Care, Fondazione Policlinico Universitario A. Gemelli IRCCS, Università Cattolica del Sacro Cuore, 00168 Rome, Italy; christian.cuomo@guest.policlinicogemelli.it (C.C.); filomena.dellasala@policlinicogemelli.it (F.D.S.); rossano.festa@policlinicogemelli.it (R.F.); rossella.garra@policlinicogemelli.it (R.G.); federica.maiellare@libero.it (F.M.); danielamaria.micci@policlinicogemelli.it (D.M.M.); domenico.posa@gmail.com (D.P.); cmaria.pizzo@gmail.com (C.M.P.); angela.pusateri@policlinicogemelli.it (A.P.); michelangelomario.spano@policlinicogemelli.it (M.M.S.); monicalucente@gmail.com (M.L.);

**Keywords:** children, neonates, anesthesia, premedication, perioperative medicine, preoperative assessment, fasting, testing

## Abstract

This review delves into the challenge of pediatric anesthesia, underscoring the necessity for tailored perioperative approaches due to children’s distinctive anatomical and physiological characteristics. Because of the vulnerability of pediatric patients to critical incidents during anesthesia, provider skills are of primary importance. Yet, almost equal importance must be granted to the adoption of a careful preanesthetic mindset toward patients and their families that recognizes the interwoven relationship between children and parents. In this paper, the preoperative evaluation process is thoroughly examined, from the first interaction with the child to the operating day. This evaluation process includes a detailed exploration of the medical history of the patient, physical examination, optimization of preoperative therapy, and adherence to updated fasting management guidelines. This process extends to considering pharmacological or drug-free premedication, focusing on the importance of preanesthesia re-evaluation. Structural resources play a critical role in pediatric anesthesia; components of this role include emphasizing the creation of child-friendly environments and ensuring appropriate support facilities. The results of this paper support the need for standardized protocols and guidelines and encourage the centralization of practices to enhance clinical efficacy.

## 1. Introduction

The challenge of pediatric anesthesia starts when we first lay eyes on our patient. The perioperative approach must be tailored to children due to their specific anatomical and physiological characteristics. Significant differences in pathophysiological, pharmacodynamic, and pharmacokinetic mechanisms, as well as human relationships, require high professional competence integrated with well-equipped facilities.

In the past decades, many papers have emphasized the protective role of provider skills. Pediatric patients are more susceptible to critical anesthesia-related incidents [1]. Prospective observational multicenter studies confirmed a relatively high rate of severe critical events during anesthesia for surgical procedures [2,3].

The risk increases in neonates, toddlers, and those with severe comorbidities. These analyses highlight the vast variability in anesthetic practice across Europe, underscoring the need for shared protocols and guidelines endorsed by local institutions [4]. Standardization and centralization have emerged as inevitable cornerstones of good clinical practice. With this approach [5], many national societies modified clinical pathways [6] and training programs [7,8] and issued recommendations [9]. 

During the preanesthesiological visit, our mindset should prioritize paying particular attention to patients and their families, who are highly interactive units. Our evaluation and practice must consider the close relationship between children and their parents and the child’s stage of development.

A careful preoperative evaluation includes a detailed medical history and an insightful physical examination to prevent significant complications and ensure a tailored anesthetic plan is drawn up by a skilled pediatric team of anesthesiologists.

The suggested timing of this evaluation is determined by demographic factors, hospital organization, and, most importantly, clinical conditions and the type of surgical procedure [10]. Hospitalization of a healthy child for preoperative evaluation should be avoided, as should day hospital stays that do not reduce costs [6]. 

The availability of structural resources is also paramount [11]. The preoperative environment, where the patient is prepared before entering the operating room, should be near the theater where anesthesia and the surgical procedure will occur. In addition to having correctly sized equipment, age-appropriate furniture, and everything needed to manage a pediatric emergency, each setting must have a child-friendly interior design. Moreover, each area should be screened or physically separated from those for adults. While support facilities might not be directly involved in anesthetic management, they are crucial to optimize the safety and comfort of infants and children. This issue should be a priority for the anesthetist.

Radiology, a laboratory, and a pharmacy should always be available, and professionals providing those services must have adequate training in pediatric applications within their fields of expertise. Appropriate premedication should be considered in order to minimize psychological trauma related to anesthesia and surgery. 

While there is no one-size-fits-all solution, processes must be controlled to ensure each child receives the most suitable anesthesia for their case.

## 2. When We Approach the Child for the First Time

### 2.1. Medical History

The preoperative evaluation should encompass a thorough review of the child’s medical and birth history, including prematurity and related complications [12]. 

A review of prior occurrences during surgery or anesthesia should also be completed, especially those involving respiratory or cardiac events. A family history should focus on anesthesia complications such as postoperative nausea and vomiting, malignant hyperthermia, or bleeding disorders that might elevate the risk of adverse events.

The preoperative assessment delves into clinical and psychosocial factors that might influence surgery timing and aims to identify underlying conditions that warrant attention or management before surgery. The evaluation classifies the patient’s American Society of Anesthesiologists risk category. However, integrating risks, like the NARCO-SS Preoperative Risk Assessment System for Children [13] or the Johns Hopkins Score Systems (JHSRCS) [14], might offer additional insights. The literature has identified preoperative clinical risk factors for perioperative adverse events, including prematurity, ASA > 3, major surgery (including cardiac, neuro, or orthopedics), urgencies, and emergencies [2,3,15,16].

The preoperative evaluation should review the patient’s and family’s medical, behavioral, and social history, previous complications related to surgery or anesthesia, and any therapies [17]. This pre-evaluation relies on an understanding of how anesthesia affects children’s normal physiology [18]. 

Every phase of the child’s growth and any deviations from typical developmental milestones should be investigated. Particularly in former preterm infants, we must pay attention to previous clinical treatment for a risk of subglottic stenosis and postoperative apnea and to different pharmacodynamic and pharmacokinetic profiles of anesthetic drugs [19]. Cardiovascular, neuromuscular, respiratory, endocrine, and hematologic factors should be considered to identify associated risks and guide clinical testing requests [20,21,22]. A careful personal or family history of malignant hyperthermia should be elicited [23] along with risk factors for rare diseases [24]. 

Preventing anaphylactic reactions primarily hinges on documenting past reactions. Thus, during the preanesthesia consultation, a comprehensive history of atopy should be obtained, and any allergies to drugs, latex, and food cross-reactions should be recorded. Allergies should be identified and documented [25]. 

Clinicians should review a patient’s behavioral and social history and involve caregivers when appropriate. Older children and adolescents should be allowed a confidential environment to discuss topics like sexuality, stress, mood symptoms, past and present trauma or abuse, and substance use, even if this aspect requires particular experience and the possible involvement of other professional figures [26,27]. Gaining a comprehensive understanding of the psychosocial dynamics of the patient better informs the operative team and prepares the child and family for the postoperative recovery period, which can be a vulnerable time, particularly after more complex procedures.

It may be necessary to communicate with the perioperative team to determine the management of therapies on the day of surgery and if any drugs should be preventively withheld due to potential interactions with anesthesia. 

Essential preoperative information plays a crucial role in preparing pediatric patients for surgery. Although children have yet to reach legal autonomy, anesthesiologists should actively engage them in decision-making, offering explanations tailored to their clinical condition. The benefits of providing clear preoperative information, possibly utilizing visual aids or pictures, have been demonstrated to decrease anxiety in both children and their parents, mitigate negative behavior in children, and enhance postoperative satisfaction [28]. 

Preoperative planning in pediatrics encompasses many of the same steps as for adults, even if there are significant differences. First, there is a close relationship with the parents and their role in every step of the decision-making process. 

Beyond the traditional history and physical examination, attention should be paid to the child’s overall development and the presence of any syndromes. Allergy to latex in children with both spina bifida and a history of multiple surgeries can be prevented by preparing latex-safe operating rooms according to international and local protocols [29,30]. Such patients should be prioritized for surgery at the start of the day to minimize exposure to aerosolized particles. They should also wear a medical alert bracelet. Signs indicating a latex-free environment should be displayed both inside and outside the operating theater, ensuring the exclusive use of latex-free materials.

Preoperative assessment guides the anesthesiologist in determining the appropriate anesthetic techniques. Regional anesthesia becomes a viable option under suitable circumstances, provided the patient shows no specific contraindications and when regional techniques enhance pain control. The decision to employ regional techniques should be based on individual considerations, weighing the risk–benefit ratio. Pediatric regional anesthetic techniques are typically administered when the patient is deeply sedated or under general anesthesia. Performing these techniques under such conditions is deemed safe and should be considered standard care [31].

Anesthesiologists must be adept at managing local anesthetics according to the patient’s age, as the metabolism of local anesthetics changes as the child grows. Although pediatric local anesthetic systemic toxicity (LAST) is rare, it is a potentially life-threatening condition that requires prompt recognition and treatment. Infants and neonates are particularly vulnerable to developing LAST, and its detection becomes challenging when the patient is under general anesthesia [32].

### 2.2. Physical Evaluation

The clinical evaluation represents a fundamental point in the correct perioperative management of the pediatric patient and should involve a comprehensive assessment of clinical status. The goal of our evaluation is to focus on identifying unrecognized conditions or analyzing risk in already-known illnesses.

During a cardiovascular examination, murmurs or rhythm abnormalities might occasionally be detected. It is not uncommon for cardiac morbidity related to congenital heart disease to arise during the first year of life. Such diseases, sometimes latent after birth, could emerge during surgery when hemodynamic changes caused by anesthetic agents, mechanical ventilation, and blood loss eventually unmask these heart defects. Echocardiography can help assess heart function and motion, and a pediatric cardiologist should always evaluate patients with suspected congenital pathologies to stratify preoperative risks correctly and optimize heart function prior to surgery [33]. An experienced pediatric cardiologist must always evaluate children with known congenital heart disease before anesthesia if a recent report is unavailable [34]. Previous surgery for correction of congenital defects should be reported and thoroughly analyzed by the anesthesia team.

It is a central issue to always examine lower airways with a standard stethoscope auscultation that might detect wheezes, rales, altered breath sounds, stridor, wheezing, or murmurs and evidence of neurologically altered breath patterns. The respiratory rate is a sensitive marker of pulmonary problems. Symptoms like nasal flaring, intercostal retractions, and pronounced use of accessory respiratory muscles all signify respiratory distress. Upper respiratory infections (URIs) presenting with fever, wheezing, or productive cough are associated with an increased risk for perioperative respiratory adverse events [35]. These conditions can frequently emerge acutely in children and may not be evident during the initial anesthetic assessment. Consequently, it is crucial to reassess them in the immediate preoperative period, as they could necessitate cancellation of elective procedures [36]. Such procedures should be rescheduled only after the inflammatory or infective condition resolves. 

Patients with neurological deficits should be studied very carefully. Muscular dystrophies, CPT2 deficiency, and congenital myopathies need special attention due to their extensive implications for anesthesia [37]. Young children with hypotonia lacking a formal diagnosis should be identified to ensure necessary precautions are taken. The lengthy diagnostic process should not be considered as a reason to delay necessary surgery.

During the physical examination, it is necessary to identify pre-existing conditions of malnutrition and dehydration to restore the child’s physiological equilibrium. Preoperative malnutrition, indeed, correlates with postoperative complications and increased hospital stay [38]. 

A physical examination is essential when planning a regional anesthetic technique. Cutaneous infections in proximity to the local anesthetic injection site serve as a contraindication to employing regional techniques. Challenges may arise in patients with preexisting neurological diseases, congenital anatomical abnormalities (such as arthrogryposis or osteogenesis imperfecta), or in those who have undergone previous surgery (such as spina bifida) [32]. In such cases, an anesthesiologist should proactively anticipate potential challenges by preparing additional equipment or considering alterations to the local anesthetic injection site or techniques.

Airway examination

A critical aspect of the physical examination involves assessing upper airway anatomy. The preoperative airway examination aims to identify physical characteristics that may indicate potential challenges with facemask ventilation or tracheal intubation. Certain features, such as limited mouth opening, restricted neck mobility, maxillary hypoplasia, or mandibular hypoplasia, should be considered warning signs. When dealing with malformations, conditions associated with decreased compliance of the submandibular space, or craniofacial syndromes, it is essential to anticipate and thoroughly investigate the potential for a difficult airway. Adequate preparation, including the provision of all necessary devices, is crucial to managing potential difficulties in airway scenarios within the operating room.

Few systematic and validated studies have been able to identify predictors of difficult mask ventilation or tracheal intubation in the pediatric population [39]. The mandibular space could be a central focus because the reduction of this space (e.g., micrognathia) restricts the space available for soft tissue displacement by direct laryngoscopy. The mentohyoid distance offers an estimate of the mandibular space, with a “normal” airway in infants requiring a minimum distance of 1.5 cm [39].

Ultrasound examinations have been employed to inspect a child’s airway, aiming to outline internal anatomical features, predict the optimal endotracheal tube size, and identify subglottic stenosis [40].

The literature indicates that children under one year of age, especially underweight ones with an ASA physical status of three or four, often face challenges with direct laryngoscopy [41].

The most comprehensive data on intubation attempts in children with difficult airways come from the multicenter Pediatric Difficult Intubation Registry [42]. An analysis of this registry, including over 1000 intubations in children with challenging airways, revealed that complications were linked to multiple intubation attempts, weight less than 10 kg, short thyromental distance, and three direct laryngoscopy attempts before the use of an indirect technique.

The significant prevalence of obstructive sleep apnea syndrome (OSAS) must also be factored into the airway management of these patients. Obstructions can occur at various levels, often causing significant anatomical distortions in the nasopharyngeal region, leading to obstructive apnea in many cases [43,44].

### 2.3. Diagnostic Tools

The preoperative evaluation aims to identify and optimize conditions that increase perioperative morbidity and mortality. Historically, pre-noncardiac surgery evaluations entailed a series of standard tests applied universally. However, these tests often do not influence perioperative management and can lead to unnecessary additional tests, potentially leading to avoidable delays in surgery and increased healthcare costs [45,46]. 

Preoperative testing for anesthesia should be required only if it is helpful to stratify risk or guide perioperative management. The decision to order preoperative tests should be based on the patient’s clinical history, comorbidities, physical examination findings, and planned surgery. According to the Surgical Risk Score, routine laboratory testing is not recommended for healthy children and adolescents undergoing low-risk and very low-risk procedures. However, patients with underlying conditions might benefit from targeted laboratory and imaging studies to assess clinical stability. 

Routine hemoglobin testing is not typically required for most elective procedures, especially when the surgical procedure is not expected to result in significant blood loss. There are insufficient data in the literature to make strict hemoglobin testing recommendations in healthy children [47,48]. A hemoglobin test is required in healthy patients without risk factors for anemia who undergo procedures with a potential for blood loss or those with specific risk factors such as hematologic disease, malnutrition, and reduced blood volume, even if asymptomatic. 

Preoperative hemostatic screening tests (PHSTs), such as platelet count, INR, and aPTT, could be contemplated only if there is bleeding risk (e.g., major surgery or tonsillectomy) and if warranted by the medical history of coagulopathy. In fact, even these indications are debatable because, in the absence of a medical history, even surgeries such as tonsillectomy should be performed without PHST. [49]. Some authors have questioned the need for a routine PHST in general surgery, suggesting that it should be performed only in specific patients with clinical abnormalities [50]. However, this approach should include a careful medical history, a specific questionnaire, and a rigorous physical examination. 

Collecting a preoperative type-and-screen or type-and-crossmatch sample is advised in preparation for potential blood transfusions, depending on the nature of the surgery and anticipated blood loss.

In a healthy patient with no medical history and without abnormalities found during a physical examination, there is no need for coagulation tests to be conducted before performing regional anesthetic techniques. However, if the medical history or physical examination indicates an increased risk of bleeding, laboratory tests should be limited to assessing aPTT, INR, and the number and functionality of platelets. If the results of the coagulation tests are positive, the anesthesiologist should refrain from using central regional techniques and opt for peripheral nerve blocks whenever possible [51].

Patients taking anticoagulation or antiplatelet drugs require careful evaluation when planning regional anesthetic techniques.

Considering the reluctance to share one’s sexual history, a pregnancy test should be suggested for all postmenarchal adolescents with confirmed sexual activity [52]. The patient and the parents should be adequately informed because of the ethical, psychological, and legal dilemmas [53]. 

The utility of a preoperative EKG should be gauged in terms of sensitivity, specificity, and predictive values. The predictive power of preoperative EKG for postoperative cardiac complications in noncardiopulmonary surgery is weak, as is its effectiveness in changing anesthetic plans [54].

In adults, various studies have shown significant variabilities in abnormal EKGs; these variabilities rarely led to changes in anesthesia [55,56]. Research on pediatric populations has drawn similar conclusions [57], leading to recommendations against routinely performing a preoperative EKG in healthy children [58].

Preoperative EKG, echocardiogram, and cardiological consultation are recommended for specific conditions and age groups, e.g., when any of the following are found: a heart murmur of doubtful interpretation, suspected congenital heart disease, OSAS, severe scoliosis, bronchopulmonary dysplasia (BPD), or neuromuscular disease. Newborns and infants up to six months of age warrant special attention due to unknown conduction anomalies such as long QT syndrome [59], Wolff–Parkinson–White syndrome [60], and congenital heart diseases [61].

A similar perspective applies to chest X-rays [62]. Systematic chest X-rays are not justified in children and must not be carried out in consideration of, above all, the biological damage from ionizing radiation exposure. They may be indicated for patients with a positive history of BPD, severe asthma, or neuromuscular disease and whenever the physical examination and clinical history suggest the need for further diagnostic workup.

### 2.4. Preoperative Therapy Optimization

Sick children are often subject to ongoing major therapies. Communicating with the operative team is essential to determine which medications should be continued or halted before surgery, especially considering potential interactions with anesthetic drugs. 

Typically, medications are continued at their usual dosage up to the day of surgery. Then, the dosage could be adjusted perioperatively to ensure adequate therapeutic levels. Any adjustment of the therapeutic plan should be shared with referring doctors to minimize risks of inappropriateness and to guarantee a smooth transition.

Epileptic patients who are on a regular dose of anticonvulsants should not stop their medication leading up to the surgery [63]. It is advisable to adhere to the regular schedule for individuals on oral therapy to prevent seizures before surgery. Anesthesia plans should consider the duration of anesthesia (to determine if additional doses are needed during prolonged surgeries) and the risk of clinically significant drug interactions, especially with traditional antiepileptic drugs [64]. Similarly, psychiatric medication might need an adjustment to keep a stable mental status and reduce the risk of adverse events in the postoperative time.

Maintaining normoglycemia is crucial for all children and neonates [65]. For diabetic patients undergoing surgery, optimal glycemic control is mandatory. If it is suboptimal, it is paramount to evaluate whether elective surgery should be delayed or, instead, a corrective therapy should be initiated as soon as possible, with close monitoring throughout the perioperative period. Diabetic patients are at increased risk of dangerous, life-threatening metabolic and systemic derangements such as ketoacidosis and hyperosmolar conditions.

The anesthesiologist should obtain specific information about patients on insulin therapy, particularly focusing on the insulin administration scheme and the type of insulin prescribed. For those on a basal-bolus regimen, the recommendation is to administer the usual dose of long-acting insulin, with a possible reduction of 20–30% if low blood glucose patterns are observed [66]. Rapid-acting insulin should be withheld on the day of surgery except in hyperglycemia cases requiring correction. In the case of insulin pump users, it is essential to document the last pump site change, discuss the surgical site plan, position the pump insertion site away from the surgical field, and verify the pump’s function and location before the procedure [67]. 

For major procedures or patients receiving neutral protamine Hagedorn insulin, additional consultation with endocrinology and coordination with the surgical team is recommended for perioperative planning [67]. 

Anticoagulant therapy also requires optimization before surgery. For patients on warfarin, bridging with a short half-life agent may be necessary. The need for bridging depends on the risk of thrombosis without antithrombotic therapy. Anticoagulation may be continued without cessation for minor procedures, while bridging is needed for most major procedures. The CHEST guidelines recommend stopping warfarin five days before the planned procedure and resuming therapy 12 to 24 h after the end of surgery, in agreement with the surgeon [68]. It is recommended to withhold unfractionated heparin for 4 to 6 h before surgery and to resume when hemostasis is under control [68].

Direct oral anticoagulant (DOAC) use in pediatric patients remains uncommon, although use in older adolescents, sickle cell anemia, or clinical trial participants does occur in clinical practice [69]. A pharmacokinetic approach to managing DOACs in the perioperative setting suggests they should be withheld for 1 to 2 days before and restarted the day after surgery [70]. Adherence to clinical trial recommendations or consultation with a pediatric hematologist should be advised for pediatric patients.

Antiplatelet agents in pediatric patients are mainly used in the cardiac population, and thus, procedural management is often specific to the procedure and the underlying reason for antiplatelet therapy. The need to withhold antiplatelet therapy depends on the risk of thrombosis in the absence of therapy and the risk of bleeding related to the procedure. In most cases, aspirin can be safely continued; however, clinically relevant, nonmajor bleeding has been observed in patients undergoing cardiac surgery [68,71]. 

For patients for whom aspirin can be safely withheld, it should be suspended 7 to 10 days before the procedure [68]. For patients on clopidogrel, it should be discontinued five days before the procedure [68,71].

Patients with a history of frequent asthma exacerbations, especially those requiring ICU admission or emergency admission and those on systemic corticosteroids, may be at higher risk for bronchospasm during intraoperative care and for postoperative adverse outcomes [72,73]. Chronic medication regimens, including inhaled corticosteroids, should be optimized [72]. The preoperative administration of a short course of oral corticosteroids and an inhaled β2-adrenergic agonist and anticholinergic agent 1–2 h before surgery can decrease airway reactivity and perioperative bronchospasm, including the response to endotracheal intubation (Table 1) [73].

Premedication with corticosteroids and antihistamines 

Preventive treatment with corticosteroids and antihistamines, either anti-H1 or combined anti-H1 and H2 antagonists, remains controversial [74]. While pre-emptive treatment has shown a reduction in the severity but not the incidence of adverse reactions to medications, it has generally proven effective in minimizing the adverse effects of nonimmune release to NMBAs, gelatine, contrasts, and vancomycin [75]. However, the clinical conditions and the pathogenic mechanism of the reaction could influence the severity of the responses. A recommended pretreatment for allergic reactions is shown in Table 2 [76].

Endocarditis prophylaxis 

After many years of widely recommended endocarditis prophylaxis, in 2007, there was a shift to only recommended its use in high-risk patient populations [77].

Experts agree that infective endocarditis is much more likely to result from frequent exposure to random bacteremias associated with daily activities than from bacteremia caused by procedures, and prophylaxis may prevent an exceedingly small number of cases of infective endocarditis [78]. The most recent guidelines suggested prophylaxis in children with congenital heart disease undergoing any procedure at risk for transient bacteremia (e.g., dental, sinus, airway, genitourinary, gastrointestinal) or when the surgical site could be contaminated (Table 3). Other cardiac conditions for which prophylaxis is reasonable are when the patient has a history of previous infective carditis and in cases where prosthetic material was used for valve repair. Patients with anatomical malformations, such as bicuspid aortic valve and mitral valve prolapse, do not require perioperative prophylaxis [78]. The optimization of preoperative antibiotic administration should always be ensured, especially in the context of major surgery [79]. 

### 2.5. The Role of Families

The lives of children are intimately connected to their caregivers [80]. Facing a stressful event such as anesthesia and surgery could be both a traumatic experience and a moment of collective maturation. During the preoperative time, anesthetists should cooperate to support children and their parents in coping with the situation to reduce anxiety levels. This takes a great deal of skill and emotional disposition. 

Factors contributing to anxiety and emotional distress in parents and children include a lack of familiarity with the surgical setting, including the medical equipment, a lack of preparation for painful procedures, and inadequate preoperative information [20]. Older children may struggle to process and verbalize their fears and anxieties [81]. Operating rooms, medical tools, and even staff in surgical attire can be intimidating. Due to all these issues, coupled with the fear of the unknown, children might react with nightmares, crying, or refusal to speak [82]. The experience can also be overwhelming for parents.

Simultaneously, parents, while attempting to remain strong, might internally wrestle with fears of complications, guilt over their perceived inability to protect their child, or concerns about postoperative recovery [83]. 

Considering sociocultural and economic factors is essential for enhancing the quality of family involvement, and a well-defined strategy should be adopted. Recognizing the uniqueness of each child is paramount, as what proves effective for one may not be suitable for another. It is crucial to observe and comprehend the specific needs of each child. For example, some children may be comfortable discussing their feelings, while others might prefer engaging in physical activities like drawing or playing.

Clarity and openness tailored to the family group during the preoperative consultation are vital factors. 

Visual aids like illustrated books, animations, or medical toys may all be effective in aiding the child in visualizing and processing the upcoming event [84,85]. Web-based tools can convey preoperative information to parents before pediatric ambulatory surgery [86]. Still, standardized research that enables further comparison across studies is needed.

Some centers offer courses or informational sessions for parents on managing preoperative anxiety [87]. These sessions provide valuable insights and an opportunity for parents to exchange experiences with others facing similar situations. Improper emotional handling can have immediate effects, such as a challenging anesthesia induction [88], and longer-term consequences. Emotional trauma might linger, influencing future medical visits or procedures. In contrast, effective handling not only ensures a smoother anesthesia process but can also establish trust in medical staff, diminishing fear of future procedures [89].

If anxiety or fear is particularly acute, professional psychological support may be beneficial. A specialized therapist or psychologist can provide tailored strategies and tools for emotion management and preparing the child and parents for the procedure [90].

Parents and caregivers are also involved in legal issues. In each country, national laws should be applied while respecting Article 24 of the UN Convention on the Rights of the Child [91]. However, disagreement about legal caregivers’ identification is increasing. Conflict might also arise between medical staff and parents. Good communication and collaboration with parents—and children, when possible—can help reduce these conflicts [92].

Healthcare professionals’ primary focus is acting for the child’s “best interests”, although the family may not understand this [93]. Healthcare institutions should utilize mediation tools and conflict management frameworks to reduce legal disputes [94]. Once again, only experienced and dedicated anesthetists will be able to deal with these crucial aspects.

## 3. The Operating Time

### 3.1. Fasting

Fasting is a standard procedure before elective general anesthesia or procedural sedation, but its metabolic effect and substantial impact on the child’s well-being must be kept in mind.

In the last few years, several national societies have adopted more liberal fasting guidelines, encouraging the avoidance of prolonged fasting times in all children scheduled for elective surgery because it is a safe procedure and protective against any metabolic derangements [95,96,97,98].

The traditional “Nil by mouth from midnight” fasting regimen disrupts normal homeostasis, leading to potential adverse intra- and postoperative effects [99]. Prolonged fasting can result in hypoglycemia, ketoacidosis/metabolic instability, electrolyte imbalance, or hemodynamic instability because of hypovolemia. Insulin resistance is also involved [100]. The latter arises from decreased insulin release and increased free fatty acids and is exacerbated during surgery due to increased stress hormone release (catecholamines, cortisol, glucagon).

Some authors highlighted that actual fasting times often exceed the minimums recommended by international guidelines [101]. The reason for this appears to be multifactorial and involves a lack of appropriate communication, operating theater delays, differing instructions, and noncompliance by parents [100]. Extended fasting not only affects physiological mechanisms but also negatively impacts the child’s psychological state, leading to increased anxiety, sadness, and distress [102].

The latest European guidelines allow healthy children to drink clear fluids up to 1 h before anesthesia induction for elective procedures (Table 4) [96]. Despite broad adoption, this recommendation is, in fact, weakly supported, due to low-quality evidence, and the literature does not provide clear indications regarding the volume of intake. Some experts also suggest that a light breakfast of solids or nonclear fluids, like cereal with milk or buttered toast with jam, may be allowed up to 4 h before anesthesia induction. Furthermore, the definition of a “light meal” remains ambiguous.

Interestingly, there is not a clear correlation between fasting interval and intragastric volume [103]. Due to limited and inconclusive evidence, experts often struggle to provide specific recommendations [99]. In these cases, anesthesiologists could perform an ultrasound assessment of gastric contents and volume, preferring a qualitative interpretation of sonographic imaging [104].

The preoperative fasting schedule should be integrated with clinical status because diseases, medications, and prematurity could modify gastric emptying. Obesity [105], diabetes [106], gastroparesis [107], or any other cause [108] of delayed emptying requires, indeed, a longer fasting time [109]. Otherwise, a standard fasting time may adversely affect global outcomes in children with dumping syndrome [110] or metabolic disease [111]. In some rare diseases, a multidisciplinary approach to define the timing of fasting and a global evaluation of food intake during the perioperative period could be useful [112].

### 3.2. Premedication

Specific attention must be paid to anxiety reduction before any intervention on the child, whether invasive or noninvasive. Not only the surgical procedure but any healthcare experience that turns out to be traumatic for the child increases the likelihood of a fear of hospitalization. Research indicates that children who are agitated in the waiting room and during induction of anesthesia often experience more significant postoperative distress [113], manifesting as irritability, delirium, increased pain sensitivity, prolonged surgical healing times, dysrhythmia, insomnia, and reduced cognitive effort [114]. Postoperative pain, behavioral changes, and sleep disorders have been reported with 3.5 times more frequency in children experiencing preoperative anxiety [115]. In particular, 67% of children develop negative behaviors on the first day after surgery, 45% on the second day, and 23% within two weeks [116]. Negative behaviors have also been reported up to six months after surgery in 20% of children and up to one year in 7.3% of children [117]. It is unclear how perioperative anxiety influences subsequent emotional and intellectual development, but negative memories of hospitalizations and anesthetic treatments may persist even into adulthood [118]. 

Some conditions are almost always associated with an anxious background [119]: very young patients; children with an inhibited, shy, and dependent temperament; repeated hospitalizations; previous traumatic experiences; and cognitive or behavioral conditions that reduce the ability to cooperate, such as those associated with the autistic spectrum. The possibility of a negative transfer of anxiety from the parents to their child must not be neglected. Thus, anxiolytic intervention requires the same rigor as performing pediatric anesthesia. The anesthetic evaluation should include a psycho-attitudinal assessment of the child to determine the most suitable premedication strategy. For anesthesiologists, this means creating optimal conditions to perform a smooth induction of anesthesia.

Over the last 20 years, various nonpharmacological soothing techniques have been explored, including the following: the presence of parents, clown and music therapy, watching videos through tablets, touring the operating rooms, and psychological support [120]. For instance, distraction techniques through watching videos have been shown to be equally effective in reducing preoperative agitation when compared to midazolam in children aged 1–12 years [121]. However, the feasibility of these interventions often clashes with the need to meet the regular turnover of scheduled patients. 

Therefore, the use of drugs remains a quick and safe route, considering that anxiolysis represents the first level of sedation in the RASS and Ramsay sedation scale [122] (Table 5).

The ideal premedication must first and foremost be easily administered, effective in achieving the clinical effect, and safe, i.e., predictable in the onset time and the appearance of adverse events.

The oral route is the most accepted by children [123]. Midazolam, the most commonly employed drug, has the advantage of manageability due to its rapid onset and short duration of action. Additionally, it has an antidote (flumazenil), which can be used, above all, to counteract the paradoxical effects affecting 1% of patients, manifested as psychomotor restlessness. However, particular attention must be paid to the different half-lives of the two drugs: midazolam has a longer half-life than flumazenil, producing a possible rebound effect [124].

Dexmedetomidine, an alpha-2 agonist similar to clonidine, is a newer drug with sedative and analgesic properties. It offers a more favorable pharmacokinetic profile and preservation of the respiratory drive, but its cardiovascular effects and slower onset require administration at least 50–60 min before mask induction. The intranasal route, with a dosage between 2 and 3 mcg/kg, is preferred for premedication using dexmedetomidine [125].

### 3.3. Preinduction Re-Evaluation

As recommended by good clinical practice and by the latest standards of care [126], preanesthetic re-evaluation requires specific considerations for the pediatric population [20,127]. The primary objective is to ensure that the patient’s status has remained unchanged since the initial assessment.

This re-evaluation, conducted immediately before entering the operating theatre, helps confirm the preoperative medical examination and identify new conditions that may increase intraoperative risks. It is crucial to reassess the child in a comforting environment, ideally in the presence of their parents, to mitigate any perioperative stress. Anesthesiologists review clinical charts to ensure the accuracy of information and execution of prescriptions. If not already in place, informed consent must be obtained. 

During preinduction re-evaluation, it is essential to inquire about the timing and type of food ingested; this information should be integrated with clinical status to assess the risk of aspiration.

Children have a higher incidence of perioperative airway adverse events (PRAEs) compared to adults. Recent viral or bacterial URIs increase the risk of PRAEs during general anesthesia [128,129]. Astonishingly, 25–45% of children scheduled for general anesthesia present with active or recent URIs during the preoperative anesthesiologist assessment in the operating room [129]. 

The risk of PRAEs remains increased up to 4–5 weeks after the event, and elective surgery should be postponed when possible [130]. Children with seasonal or viral rhinitis who do not have fever or lower respiratory signs or symptoms may undergo general anesthesia as per the anesthesiologist’s judgment. 

Asthma, another evolving risk factor, requires careful assessment during the preinduction re-evaluation [131]. The presence of wheezing on lung auscultation should alert the anesthesiologist. Elective surgery should not be performed in children with uncontrolled asthma and should be rescheduled after optimizing therapy. Notably, children with both uncontrolled asthma and URIs face a significantly heightened risk of respiratory complications.

Due to all these observations, it is advisable to always examine the pediatric patient at the time of hospital admission.

## 4. Conclusions

This review explores evidence-based recommendations concerning preoperative management, emphasizing aspects uniquely pertinent to the pediatric domain. Although pediatric anesthesia is considered by many to be a subspecialty, we may be faced with a discipline with its own identity and uniqueness. 

The pediatric anesthetist must have adequate clinical skills. Likewise, they must know how to interact with young patients and their parents, understand their fears and needs, and adapt their way of speaking and interacting. Anesthesia and the operating theatre environment are complex systems involving human–machine and human–human interactions [132]. 

An environment where each professional figure can demonstrate competence, empathy, and conscientiousness reassures parents that their child will be well-handled and safe. This parent–physician interaction will facilitate the understanding of information regarding procedures, risks, and adequate preparation, such as preoperative fasting. Furthermore, it contributes to the collection of an accurate medical history and physical evaluation, keeping the patient’s well-being as the central target. Taking care of pediatric patients requires competence, patience, and humanity. Teamwork and professional experience grow at the same time. The primary goal is always safety but without sacrificing the simultaneous goals of reducing hospitalization time, diagnostic tests, and unpleasant interactions.

## Figures and Tables

**Table 1:** Preoperative corticosteroid regimen.

Corticosteroid	Dosage	Days Prior to Surgery
Prednisone	1 mg/kg/die (max 60 mg/die)	3–5
Dexamethasone	0.6 mg/kg/die (max 16 mg/die)	2
Methylprednisolone	1 mg/kg	Single dose 48 h before surgery

**Table 2:** Allergic premedication.

Drug	Dosage	Time
Prednisone	0.5–0.7 mg/kg (max 50 mg)	24 h and 1 h before surgery
Cetirizine	2.5 mg up to 6 years 5 mg over 6 years	24 h and 1 h before surgery

**Table 3:** Endocarditis prophylaxis.

	Drug	Oral Dosage	IV/IM Dosage
Standard regimen	Amoxicillin	50 mg/kg	
Patients unable to take oral medication	Ampicillin		
	Cefazolin		50 mg/kg
	Ceftriaxone		
Patients allergic to penicillin	Cephalexin	50 mg/kg	
	Clindamycin	20 mg/kg	
	Azithromycin	15 mg/kg	
	Clarithromycin	15 mg/kg	
Patients allergic and unable to take oral medication	Cefazolin		50 mg/kg
	Ceftriaxone		50 mg/kg
	Clindamycin		20 mg/kg

**Table 4:** Fasting time regimen in children.

	Timing (Hour)			
	1 h	3 h	4 h	6 h
Food/drink	Water	Breast milk	Formula milk	Solid food
	Coffee		“Light breakfast”	
	Tea			
	Pulp-free juice			

**Table 5:** Premedication drugs.

Drugs	Oral Dosage	Intranasal Dosage
Midazolam	0.5–0.8 mg/kg	0.2–0.3 mg/kg
Ketamine	5 mg/kg	2–4 mg/kg
Clonidine	4 mcg/kg	3–4 mcg/kg
Dexmedetomidine	3–4 mcg/kg	2–3 mcg/kg
Fentanyl		1.5 mcg/kg
Sufentanyl		0.5 mcg/kg

## Data Availability

Not applicable.
